# The complete plastid genome of Chinese cinnamon, *Cinnamomum aromaticum * Nees (Lauraceae)

**DOI:** 10.1080/23802359.2019.1685414

**Published:** 2019-11-05

**Authors:** Peiwu Xie, Shanzhi Lin, Qiang Lai, Huiming Lian, Jielian Chen, Qian Zhang, Boxiang He

**Affiliations:** a Guangdong Provincial Key Laboratory of Silviculture, Protection and Utilization/Guangdong Academy of Forestry, Guangzhou, China;; b Beijing Advanced Innovation Center for Tree Breeding by Molecular Design, College of Biological Sciences and Biotechnology, National Engineering Laboratory for Tree Breeding, Beijing Forestry University, Beijing, China;; c Key Laboratory of Plant Resources Conservation and Sustainable Utilization, South China Botanical Garden, Chinese Academy of Sciences, Guangzhou, China

**Keywords:** China, chloroplast genome, phylogeny, laurel family

## Abstract

*Cinnamomum aromaticum* has long been recognized and cultivated in tropical and subtropical Asia for their aromatic bark to produce cinnamon. We reported for the first time the complete plastid genome of *C. aromaticum* and reconstructed its phylogenetic position. The complete plastid genome is 152,754 bp in length with a quadripartite organization: a large single copy (LSC) region of 93,706 bp and a small single copy (SSC) region of 18,916 bp. Each of the two inverted repeat regions (IRa and IRb) is 20,066 bp. We recovered 128 functional genes, including 84 protein-coding genes, 36 tRNA genes and 8 rRNA genes. The phylogenetic analysis suggested that *C. aromaticum* and two samples of *C. camphora* forms a strongly supported clade, which is sister to another cinnamon species of *C. verum* native to Sri Lanka with strong ultrafast bootstrap support.

Several species of the genus *Cinnamomum* in the Lauraceae family, e.g., *C. aromaticum* Nees, *C. verum* J. Presl*, C. citriodorum* Thwaites, have long been recognized for their economical importance as source of spice. Of these species*, C. aromaticum* (= *C. cassia* (L.) Bercht. & Presl. and *C. cassia* D. Don), also known as Chinese cinnamon or Chinese cassia originated in southern China, is the most widely cultivated species in tropical and subtropical Asia for their aromatic bark to produce cinnamon (Wang and Tang 2006; Wu et al. 2008; Huang et al. 2016). For a better understanding of the plastid genome characterization and its phylogenetic relationships with other *Cinnamomum* species, we generated the plastid genome of *C. aromaticum* using genome skimming method.

The fresh leaf tissues of *C. aromaticum* were collected from South China Botanical Garden, Guangzhou, China (113.36°E, 23.19°N). Voucher specimens (XPW504) were deposited in the IBCS. We isolated the whole genomic DNA using a modified CTAB method (Doyle and Doyle 1987). We fragmented the isolated total genomic DNA into 300-500 bp in length to construct libraries following the manufacturer’s manual (Illumina). Paired-end (PE) sequencing was conducted on the Illumina HiSeq X-Ten instrument at Beijing Genomics Institute (BGI). We used GetOrganelle pipeline (Jin et al. 2018) to assemble the plastome. The GetOrganelle automatically recruits plastid-like reads by using Bowtie2 (Langmead and Salzberg 2012), and assembled the filtered reads using SPAdes (Bankevich et al. 2012). We generated the complete circular chloroplast genome by Bandage (Wick et al. 2015). We employed Plastid Genome Annotator (PGA) (Qu et al. 2019) and Geneious v11.0 (Kearse et al. 2012) to annotate the plastome and to verify the accuracy of the assembly. The annotated plastome has been deposited in GenBank (MN173819).

To reconstruct the phylogenetic tree of *C. aromaticum*, we included 19 plastid genomes in previous publications and unpublished data in GenBank (Figure 1) (Song 2016; Wu et al. 2016; Chen et al. 2017, 2019; Song et al. 2017; Zeng et al. 2018). We aligned the data matrix using MAFFT v.1.3.7 (Katoh and Standley 2013) with default parameters. The maximum likelihood tree was build in IQ-tree (Trifinopoulos et al. 2016) using models recommended by ModelFinder (Kalyaanamoorthy et al. 2017). The branch supports were estimated using 1000 integrations of ultrafast bootstrap (Hoang et al. 2018).

**Figure 1. Phylogenetic position of Cinnamomum aromaticum inferred from likelihood method based on 19 complete plastome sequences from Lauraceae. Numbers on the nods are nanparametric bootstrap values from 1000 replicates.**
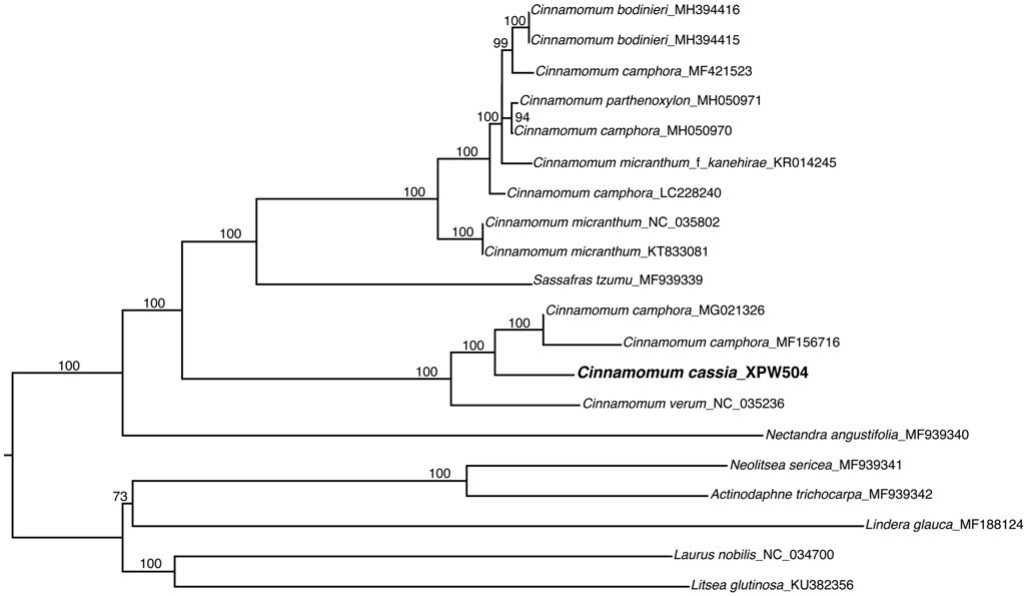


The complete plastid genome of *C. aromaticum* was 152,754 bp in length and showed a typical quadripartite organization: a large single copy (LSC) region of 93,706 bp and a small single copy (SSC) region of 18,916 bp, respectively. These two regions were separated by two inverted repeat regions (IRa and IRb), each of 20,066 bp in length. A total of 128 functional genes were recovered, consisting of 84 protein-coding genes, 36 tRNA genes and 8 rRNA genes. The phylogenetic analysis suggested that *C. aromaticum* and two samples of *C. camphora* (L.) J. Presl forms a strongly supported clade, which is sister to another cinnamon species of *C. verum* native to Sri Lanka with strong bootstrap support. This study demonstrated the potential power of genomic data in resolving the phylogenetic relationships among cinnamon species and in answering questions regarding their genetic diversity.
